# Cancer risk and mortality in patients with solitary Peutz–Jeghers polyps

**DOI:** 10.1093/gastro/goad056

**Published:** 2023-09-22

**Authors:** Anne Marie Jelsig, Laus Wullum, Lilian Bomme Ousager, Johan Burish, Tine Plato Kühlmann, John Gásdal Karstensen

**Affiliations:** Department of Clinical Genetics, University Hospital of Copenhagen, Rigshospitalet, Copenhagen, Denmark; Omicron Aps, Copenhagen, Denmark; Department of Clinical Genetics, Odense University Hospital, Odense, Denmark; Human Genetics, Institute of Clinical Research, University of Southern Denmark, Odense, Denmark; Gastrounit, Medical Division, Copenhagen University Hospital—Amager and Hvidovre, Hvidovre, Denmark; Department of Pathology, Copenhagen University Hospital, Herlev, Denmark; Department of Clinical Medicine, University of Copenhagen, Copenhagen, Denmark; Department of Clinical Medicine, University of Copenhagen, Copenhagen, Denmark; Danish Polyposis Registry, Gastrounit, Copenhagen University Hospital—Amager and Hvidovre, Hvidovre, Denmark

## Introduction

**Figure 1.**
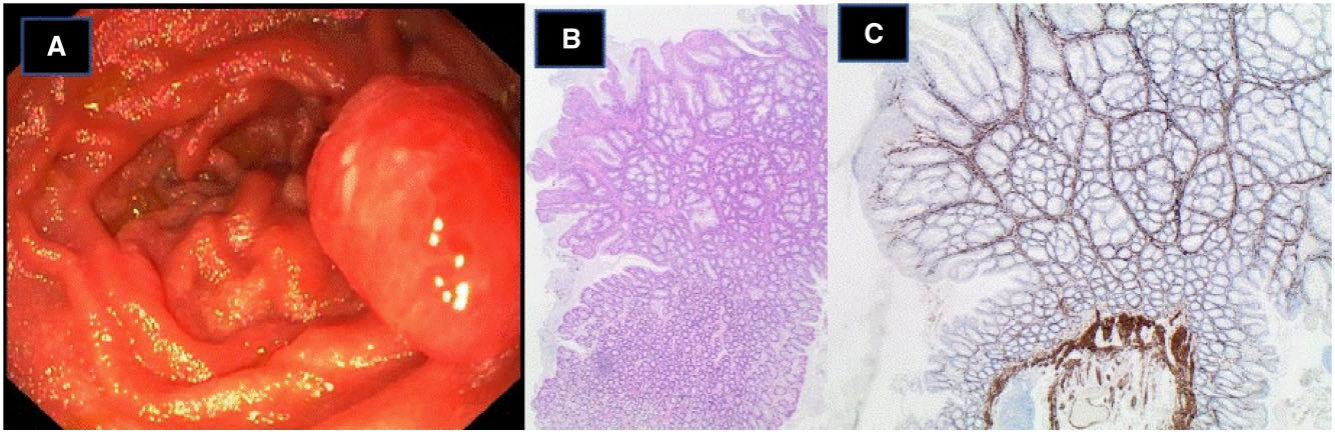
Macroscopic and microscopic appearance of Peutz–Jeghers polyps. (A) Peutz–Jeghers polyp in the small intestine. (B) and (C) Characteristic microscopic morphology of a Peutz–Jeghers polyp in the colon. Peutz–Jeghers polyps are hamartomatous mucosal polyps with a central core of branching smooth muscle (accentuated by smooth muscle staining for Caldesmon in (C)) often associated with nodular arranged normal mucosa native to the site of origin.

Solitary Peutz–Jeghers polyps are hamartomatous lesions in the gastrointestinal (GI) tract. They are rare and can be diagnosed in both children and adults (Figure 1A). Microscopically, Peutz–Jeghers polyps can have a characteristic morphology, making it possible to distinguish them from other polyps (Figure 1B and C).

It is pivotal to distinguish patients with solitary Peutz–Jeghers polyps from patients with Peutz–Jeghers Syndrome (PJS). PJS is characterized by an increased risk of several types of cancer and multiple GI polyps, and patients are recommended for lifelong surveillance [1–3]. A clinical diagnosis of PJS is based on the occurrence of a minimum of two Peutz–Jeghers polyps, often in addition to characteristic mucocutaneous pigmentations [4]. Pathogenic germline variants can be detected in the serine/threonine protein kinase 11 gene *STK11* in >90% of patients fulfilling the clinical criteria [5].

To exclude PJS, patients diagnosed with a Peutz–Jeghers polyp are recommended to have pan-enteric endoscopic evaluation, a clinical examination, and family history check for PJS-related features. Even if PJS is unlikely, it may be difficult to decide on whether the patient should be recommended for follow-up endoscopy. In general, a solitary lesion is considered to be without neoplastic potential, but this assumption is based on poor evidence with only a few non-comparative studies [6, 7].

In this study, we retrieved data on all patients with a solitary Peutz–Jeghers polyp over a 20-year period and compared them to a cohort of matched controls. We hypothesized that a solitary Peutz–Jeghers polyp does not increase the risk of cancer or mortality.

## Patients and methods

Patients with a solitary Peutz–Jeghers polyp were identified from the Danish National Pathology Register (Hvidovre, Denmark), in which all Danish histopathological reports are registered. Using the Danish version of the Systematized Nomenclature of Medicine, we identified patients diagnosed with “hamartomatous polyp” (M75630) or “Peutz-Jeghers polyp” (S54320) from 1 January 2000 to 31 December 2019. Patients of all ages were included. All known PJS patients in Denmark were excluded [5]. The study was approved by the Danish Data Protection Agency (P-2020–560).

Patients with a solitary polyp were defined as a patient with no other hamartomatous Peutz–Jeghers polyp and who was not known to have PJS according to our previous study [5].

An experienced gastrointestinal pathologist reviewed all pathology reports. Only cases in which a characteristic microscopic Peutz–Jeghers morphology was described were included.

The control cohort was created by Statistics Denmark using the unique social security number given to all Danes at birth. Cases were matched with 50 controls on year of birth, sex, and zip code at birth. The incidence of cancer was retrieved from the Danish Cancer Registry, which includes all cancer diagnoses in Denmark since 1943. Additionally, the date and cause of death were extracted from the National Cause of Death Registry.

Follow-up began on the day on which a Peutz–Jeghers polyp was diagnosed and ended on the date of emigration, death, loss to follow-up, or end of study (31 December 2019). The incidence of cancer was estimated by dividing the observed counts by the person-years at risk and is presented with 95% confidence intervals (CIs). The cancer incidence ratio and mortality ratio were calculated. The statistical software R version 4.2.1 (R Foundation for Statistical Computing, Vienna, Austria) was used for all statistical analyses.

## Results

We identified 49 patients during the 20-year study period, meaning that 2–3 patients were diagnosed with a solitary Peutz–Jeghers polyp per year in Denmark (population 5.6 million). Most patients were male (71%) and the median age at diagnosis was 55 years (p25–p75, 38–69 years). Most polyps were found in the lower GI tract (60%). For patients with a polyp in the upper GI tract, the median age was 27 years.

Some patients were not eligible for analysis because no matched controls for them could be found. We were able to include 37 patients in our study. The number of controls was 1,861. The median follow-up time was 4.7 years (range, 0.0027–18.3 years) for cases and 5.48 years (range, 0.06–18.3 years) for controls.

Amongst cases, three patients (8%) developed cancer, of whom two were identified in the same year as the polyp was diagnosed. In the control group, there were 110 cases (6%) of cancer. The cancer incidence ratio was 1.45 (95% CI, 0.45–4.58; *P *=* *0.52). Four patients (11%) amongst cases and 111 (6%) amongst controls died during the follow-up time. The mortality rate ratio was 1.75 (95% CI, 0.72–4.27; *P *=* *0.22).

## Discussion

In this study, we compared patients with solitary Peutz–Jeghers polyps with matched controls. We failed to find any statistically significant increased risk of cancer or increased risk of mortality. Our results are coherent with the results from two previous studies, including 51 patients with solitary Peutz–Jeghers polyps with a mean follow-up of 3.8 years [6] and 8 patients with a median follow-up of 11.5 years [7]. The results support that follow-up endoscopy is not needed for patients with solitary Peutz–Jeghers polyps. In comparison, patients with PJS had an increased risk of various types of cancer, including GI, breast, pancreatobiliary, and gynecological cancers. Thus, the cumulative cancer risk has been reported to be from 47% to 90% at age 60–70 years in cohort studies [4].

This study also emphasizes that solitary Peutz–Jeghers polyps are rare. Subsequently, it was difficult to identify common features in this patient group. However, as also found in the above-mentioned studies, most patients were male, the mean age at diagnosis was >50 years, and most polyps were localized in the lower GI tract.

The strength of the study is that data coming from the nationwide Danish registers included matched controls and that the results were long-term. A limitation is that details of endoscopic follow-up, clinical examination, and genetic analyses on the included patients were missing. Thus, we do not know how the risk of PJS has been addressed. In Denmark, however, it is recommended that all patients with a solitary Peutz–Jeghers polyp are offered pan-enteric examination. Patients are also referred for genetic counseling to access family history and—in most cases—to initiate genetic testing for *STK11*. In our study, we excluded patients with known PJS beforehand and, given our previous study [5], the knowledge of patients with PJS in Denmark is quite robust. The small sample size may also result in a type II error.

## Conclusions

We did not find an increased risk of cancer or mortality in patients with solitary Peutz–Jeghers polyps. Thus, our study supports the current clinical practice of not recommending follow-up endoscopy, given that PJS has been excluded.

## Authors’ Contributions

A.M.J.: conceived and designed the project; interpreted data; drafted the manuscript. L.W.: analysed data and performed statistical analysis; critically reviewed the manuscript. L.B.O.: critically reviewed the design, data, and manuscript. J.B.: critically reviewed the design and data; critically reviewed manuscript. T.P.H.: experienced GI pathologist, critically reviewed data/manuscript. J.K.G.: supervised design and interpreted data; critically reviewed manuscript. All authors read and approved the final manuscript.
